# Hospital Management and Methods of Raising Income

**Published:** 1888-01-07

**Authors:** A. W. Webster

**Affiliations:** Chairman of Workmen's Governors, Sunderland Infirmary


### Hospital Management and Methods of Raising Income.

By A. W. Webster, Chairman of Workmen's Governors, Sunderland Infirmary.

Hospitals are now established institutions in our country, and, to a more limited extent, in almost every civilised community in the world. The philanthropic and Christian spirit that prompted their establishment is entitled to the highest praise. Possibly no institution ever founded and supported by rich and generous benefactors has been the means of alleviating more real pain among suffering humanity. Just as imminent danger breaks down all barriers of class and etiquette, so pain and suffering seem to level mankind to a common platform of sympathy. The very confusion of tongues intended by an All-wise Creator and Preserver to separate man from man, seems to be suspended in the moments of agony, for the moan or cry of pain is the same when it comes from a Greek, Turk, Russian, Englishman, or any other human being on the face of the earth. There is no denying the fact that our hospitals in the past have been almost exclusively founded, supported, aud managed by the upper and wealthier classes of society. All credit is due to such disinterested labour and sacrifice on their part, but I venture to say that God never provided them with a sphere of usefulness more capable of developing their own spiritual natures, or of giving them more downright, heartfelt satisfaction. The mere knowledge that their money and their work had been the means of easing the pain, brightening the lives and prolonging the existence of many of their less fortunate fellow-creatures, ought to be ample reward. I am far from saying that the upper, or any class, has approached the complete performance of its duty in this direction, for so much remains to be done that the liberality and benevolence of the general community will find plenty of scope for operation. After all, this support has only been given by the "select few" of the monied class, and the cry is the same from Land's End to John O'Groats—"Our hospitals need more money!" Personally, I believe most sincerely that the money will be forthcoming as soon as the constitution and management of our hospitals are re-cast to harmonise with the democratic tendency of the age.

Probably hospitals will always be institutions likely to receive the generous benefactions and active support of our best and richest men; but this is no reason why the class for whom they exist should not be encouraged, in every possible way, to assist in their maintenance. Facts bear me out when I say that, with few exceptions, the working classes have been ignored in hospital management, and this being the case, can we wonder at the meagre support given by them to such institutions? Hospital managers, under the influence and pressure of the subscribers to their funds, have forced far more of the charitable element into hospital life than is necessary or desirable. A few weeks ago the editor of this journal commented on the sturdy independence of our northern workmen. True as this may be, comparatively speaking, I see much room for improvement throughout the length and breadth of the country. We will, however, do well, in attempting to judge of the existence or non-existence of this trait of character, to remember that if the lower classes have failed to develop it so fully as their brethren in the higher social scale, it is because they have neither had the opportunity nor environment for its cultivation. Many forces are at work which tend to destroy this principle, but as I am only now dealing with it in its relation to hospital management, I must confine myself to narrow limits. Experience has taught the managers of all charitable institutions that a considerable proportion of the applicants for assistance seem to be actuated by no higher principle than that of "Get what we can." I am afraid such conduct will always be found, in a more or less exaggerated form, on this side of the Millennium; but we must not hastily condemn the working class as a body because of these exceptions. Many of our highest spirited and most independent and accomplished men have sprung from the ranks of the poor, and the honest struggles to overcome poverty, with its attendant evils, coming under my own observation deserve to be ranked with some of the most heroic deeds history can produce. I have too much personal knowledge of the poor classes not to be conscious of their profligacy and vice, but I have yet to learn that this is confined to them in particular. Most thoughtful men will admit that there are hundreds round their very doors who find it utterly impossible to make adequate provision for times of severe sickness, and it is specially for such people that hospitals exist. Notwithstanding the proportion of very poor, who perhaps could hardly give a single penny to a hospital fund without robbing some child of its breakfast, the great mass of the working class is able and willing to support such institutions.

I am not speaking without experience when I say they will do it handsomely and enthusiastically as soon as hospital managers and governors shake off their somewhat antiquated ticket and patronage systems of admission, and adopt broad and liberal forms of management. I can conceive of no method more calculated to destroy the true independence of any man than that of compelling him in his dire necessity to go begging from one subscriber to another for the privilege of medical treatment. Hospitals founded and managed in the true spirit of philanthropy are always free hospitals, the only qualification for admission being absolute necessity on the part of the sufferer, and I think the time has arrived when the public in general, and subscribers to hospital funds in particular, might safely trust the gentlemen who sit on the various committees to carry this arrangement out with care and discretion. The idea that the spirit of philanthropy can only be found where there is a "long purse" to support it, needs modifying, and I feel sure that the election of a few representative working men on the managing boards of our hospitals would do much to popularise the institutions and to prove that the spirit of philanthropy and independence existed in their class in high degree, only waiting for the opportunity of asserting and maturing itself in spheres of usefulness.

I have no intention of pleading for the recognition of a class, as such, in hospital management, my only desire being to point out that much of the charitable element may be removed by the adoption of judicious changes, embracing nothing more radical than that of asking the brotherly co-operation of all classes in the great and noble work of helping suffering humanity. I feel it almost superfluous to say anything as to the capacity of workmen for occupying such positions, it having been practically demonstrated over and over again. Several infirmaries, on a small scale, in the north of England are entirely managed and maintained by the working classes alone, and although this may never be altogether possible with our larger hospitals, I will endeavour to show in future articles how a very large proportion of income may be derived from the class by whom our hospitals are constantly filled.

Although I have pronounced "Free Hospitals" as those most likely to command support from all classes, it must be obvious that efficient and economic management, devoted and affectionate nursing, and skilled and attentive medical supervision are also imperative, to secure permanent popularity. Efficient and economic management, in this age of statistical manipulators and loquacious orators, has become a somewhat hackneyed phrase as applied to charitable institutions, yet I am convinced that many of our hospital committees have failed to balance the two as delicately and satisfactorily as might be done with a little more thought and care. Efficiency must always be maintained, as it often means the difference between life and death, but a cursory glance through some recent hospital reports will convince any unprejudiced reader that it has occasionally been purchased at a slightly extravagant price. The only economy which most people, and especially workmen, demand in hospital management is fair work for fair wages from paid officials, and a rigid prevention of waste in food, drugs, stimulants, etc.

To show the different methods of management I may cite my personal experience in connexion with two large infirmaries. The first was not a free hospital, the only cases admitted without tickets being those severe and sudden accidents which always occur in thickly-populated manufacturing districts, and even for many of these tickets were forthcoming. The economic rules of this establishment compelled patients to supply their own cutlery, as well as butter and other small things, which the most rigid economist would consider very necessary in his own household. I have often been pained at seeing many of the patients (whose delicate condition required better consideration) compelled day after day to partake of dry bread to breakfast and tea, and, worse still, having to eat their meat and potatoes like African savages. Rude and uncultivated as some were, they refused to resort to the use of their finger and thumb, and preferred waiting till some of their more favoured fellow-patients could dispense with the highly-prized table appliances. Need I say that by this time many made but a poor attack on the cold and unpalateable mess on their plates. This economy, so called, was carried on side by side with waste and extravagance. The manner of serving the food without the slightest inquiry as to whether the patient could or would eat one half, or any part of it, resulted in about one fourth of the rations served for dinner finding its way to the pig's trough. The resident staff of the institution was at the same moment living beyond the limits of economy, and in some things bordered on indulgence and extravagance.

The second was a free hospital, the only qualifications for admission being sickness and poverty. Everything necessary to the patient's comfort was found within the institution. Instead of dry bread to breakfast, butter, eggs, and other nutritious accessories were supplied. The patient's appetite and the doctor's directions alike guided the careful nurse to administer the proper quantity and quality. Waste was brought down to a minimum. The resident staff practically lived on the same diet as the patients. The practical result of the management of the first hospital, is seen in its reports issued for several years back, which show a continual falling off in the number of subscribers, a process which was gradually though surely carrying it to bankruptcy. I am glad that those interested in the hospital referred to have recently seen the error of their ways, and as some necessary reform has now been carefully introduced, and admission declared free, its popularity is already advancing with giant strides, and its present year's income is on a fair way to exceed its past by over £2,000. The management of the second hospital referred to was relatively cheaper than the first, and it commanded the respect and admiration of all. Its committee embraced men of every station and creed. Its tabulated accounts show the superiority of its systems. Its efficiency and economy will rank with the best hospitals in the kingdom, and its popularity is best seen in its continual enlargement.

*(To be continued.)*

